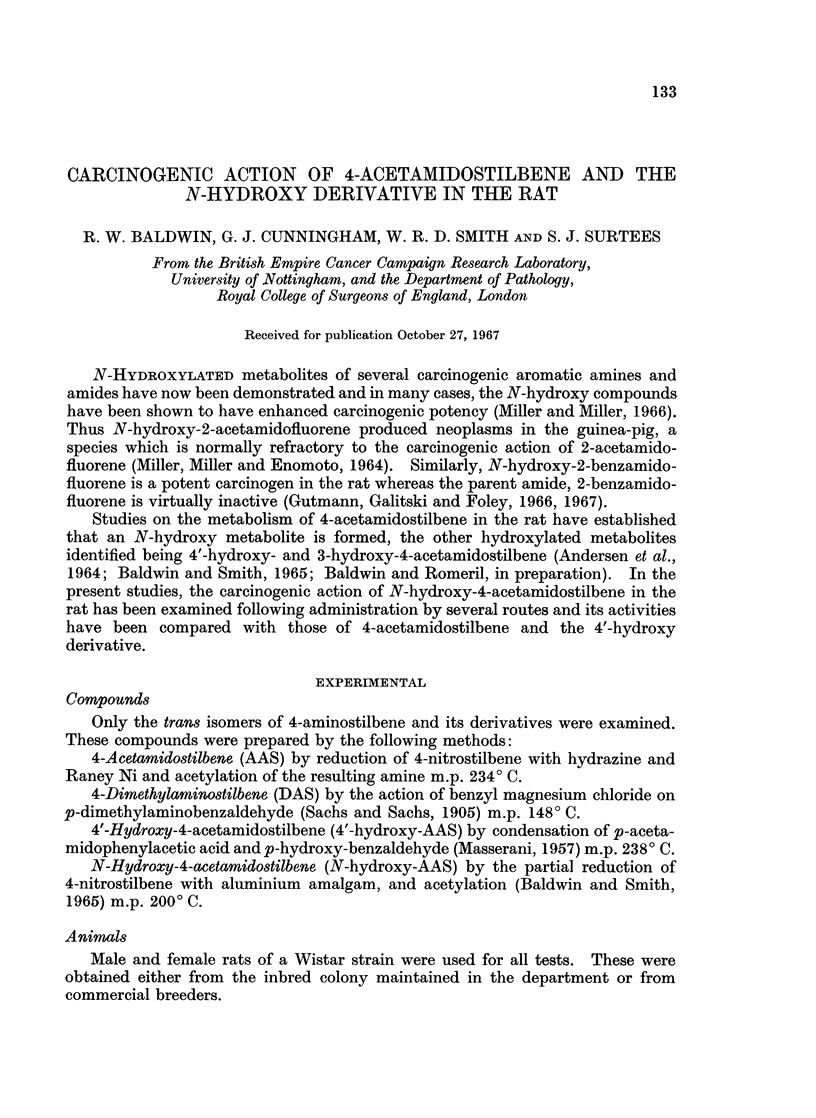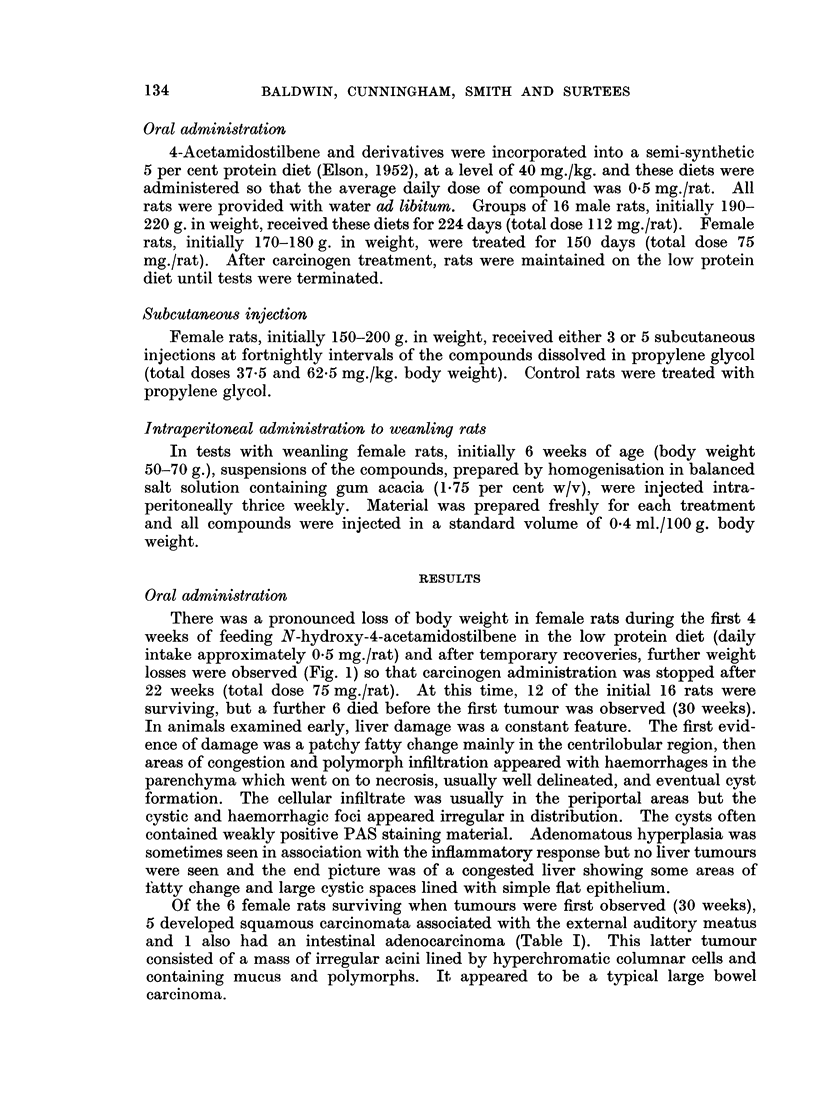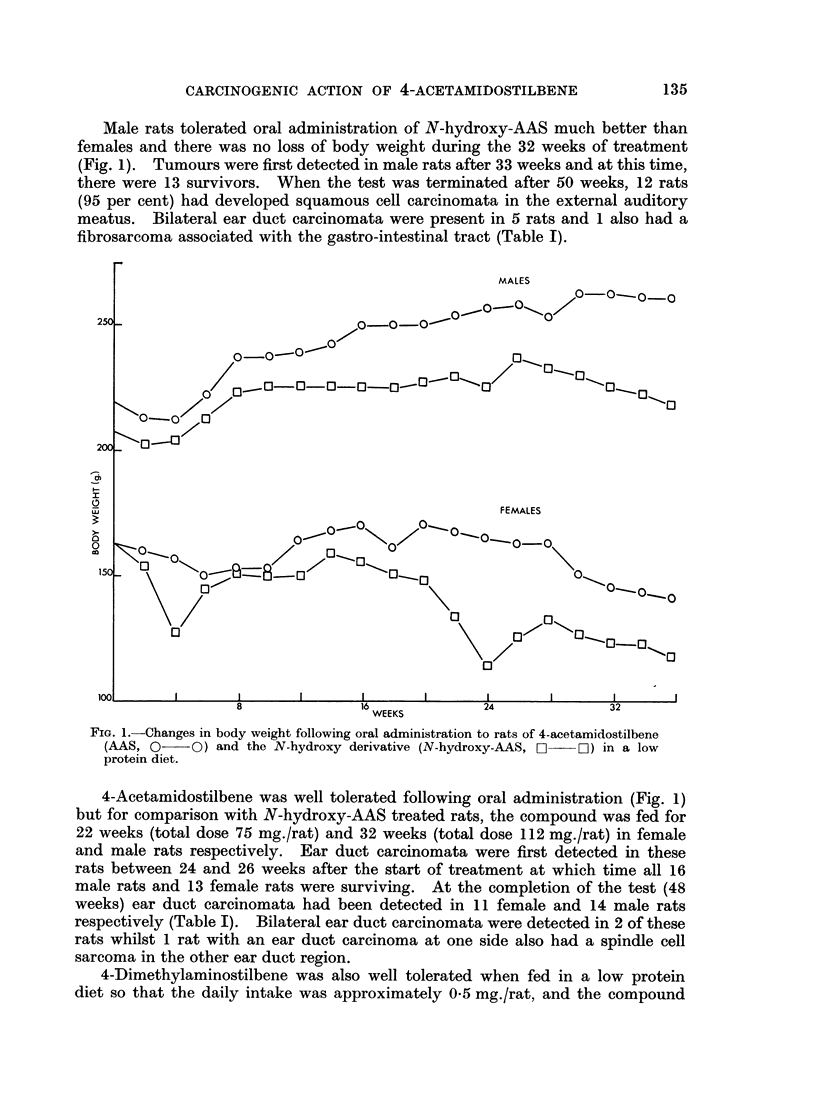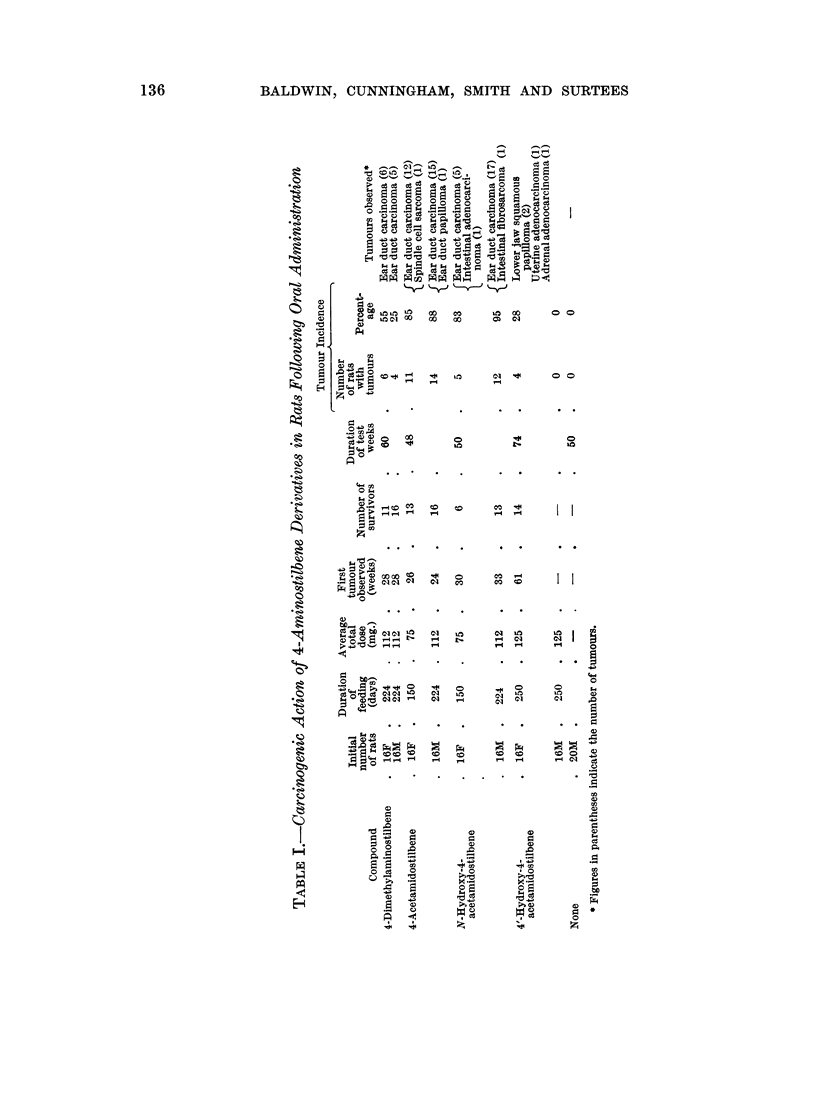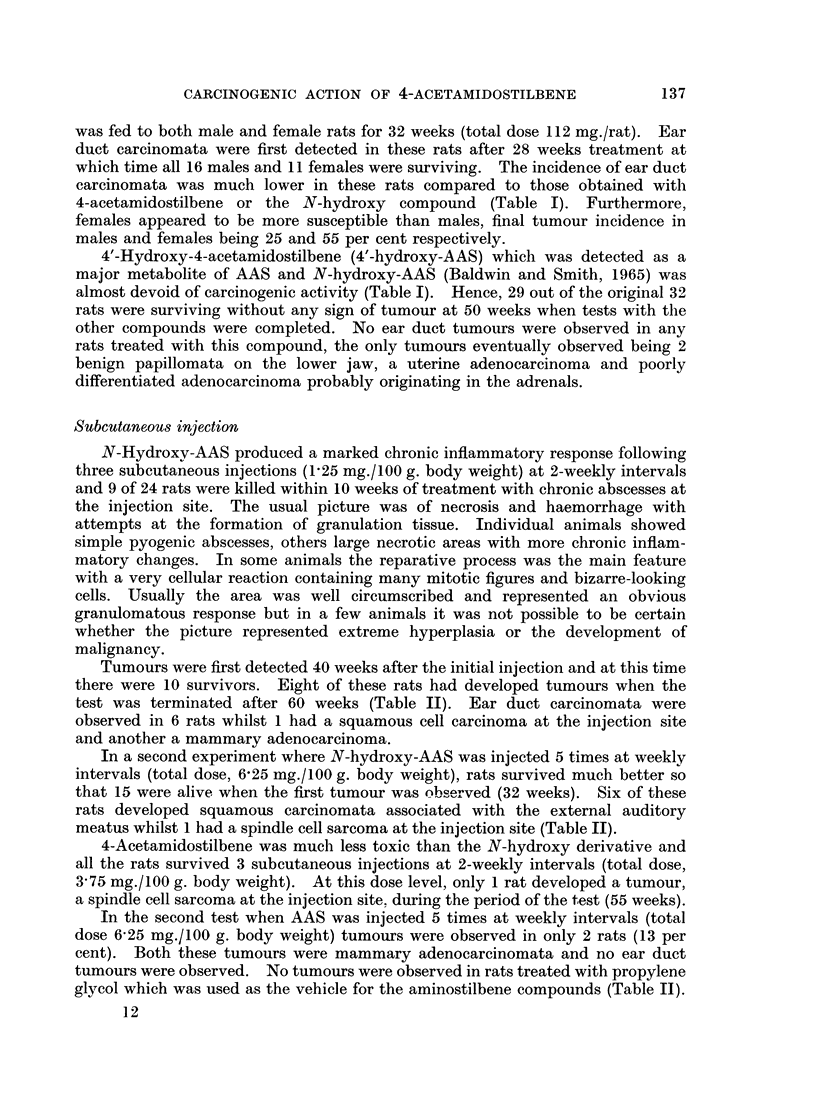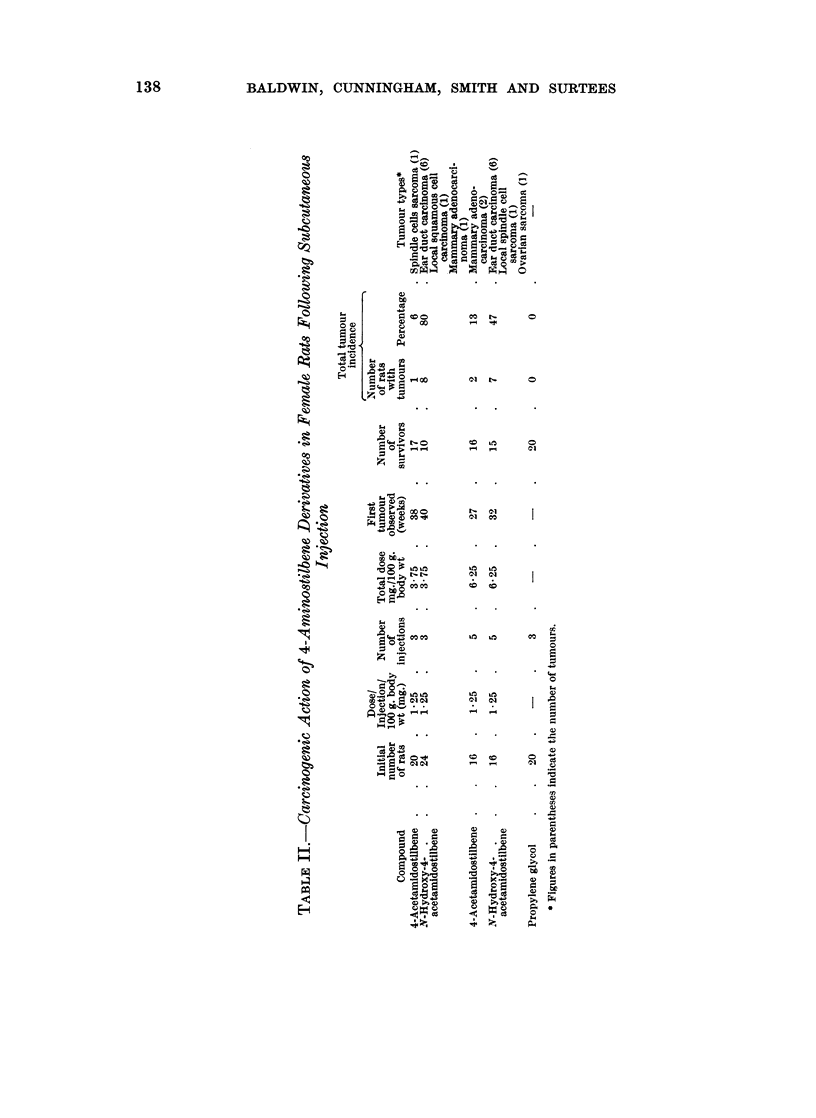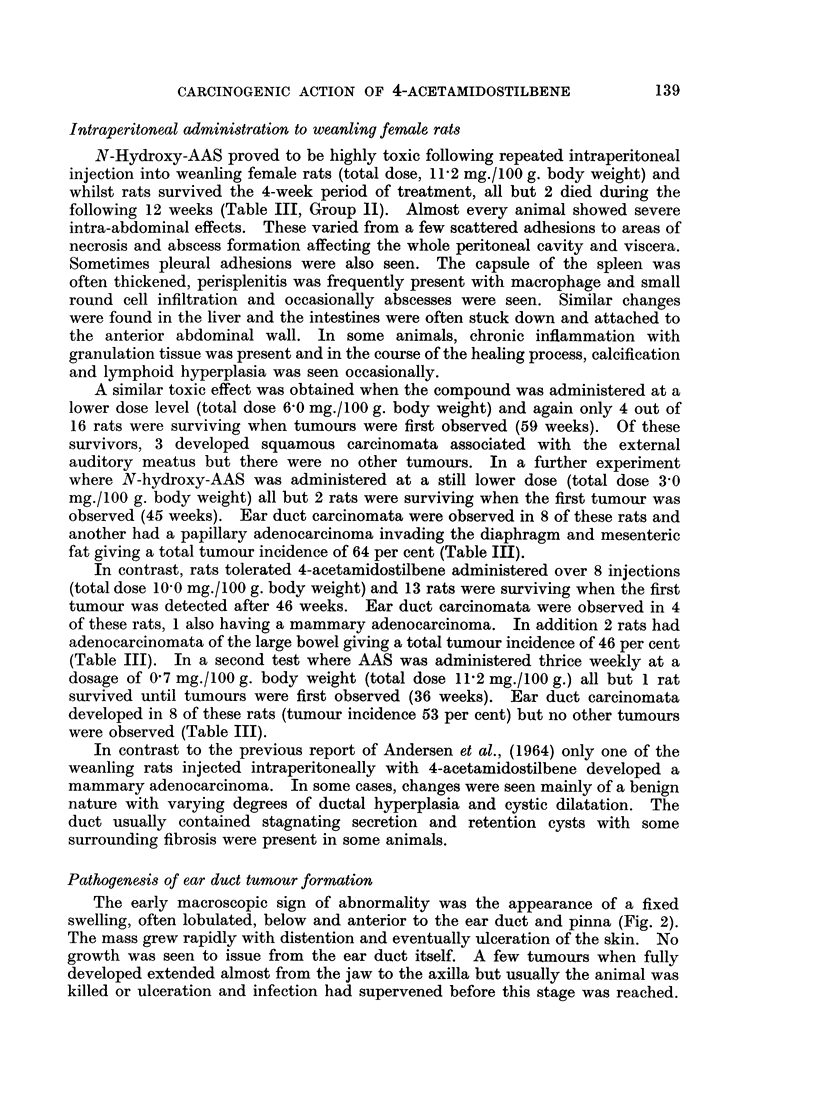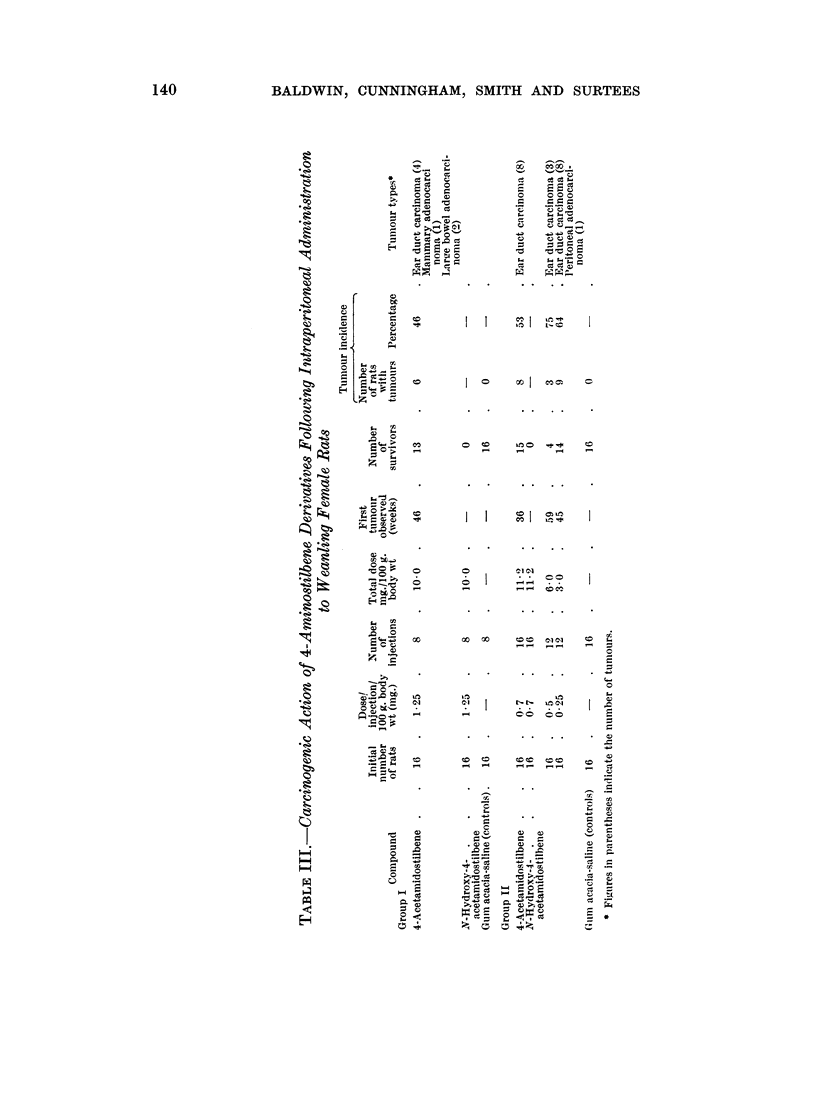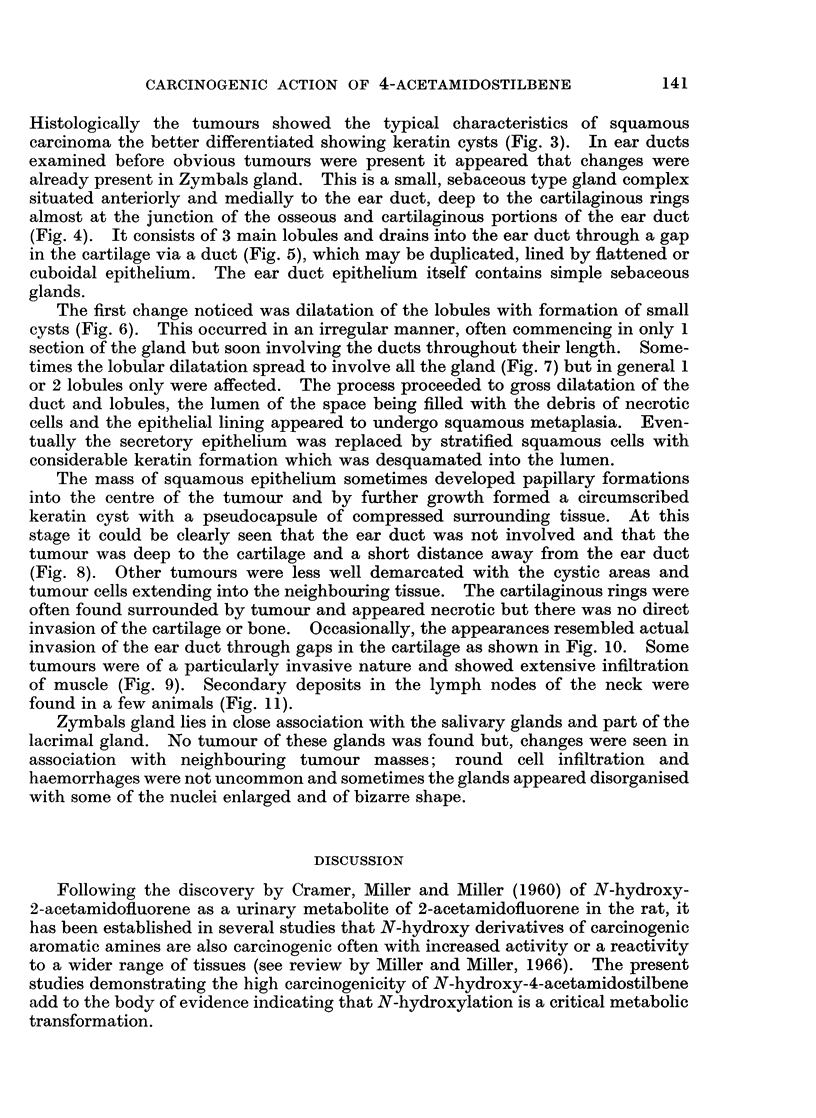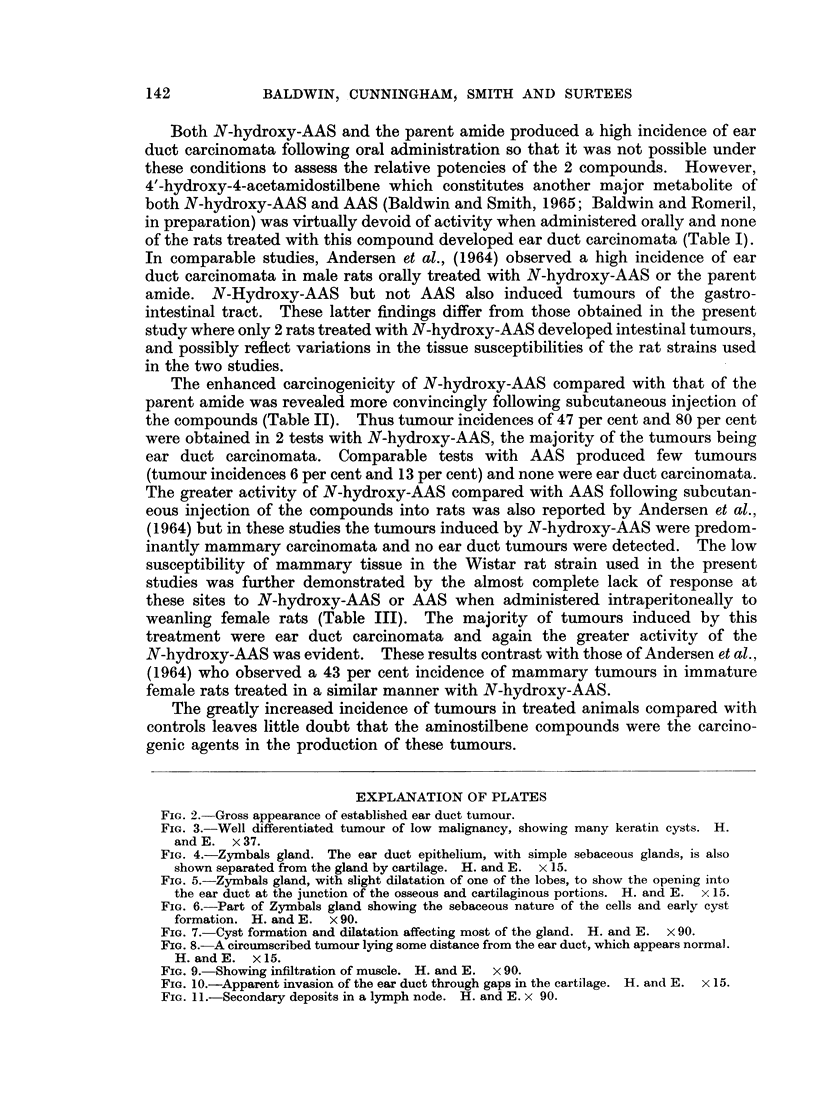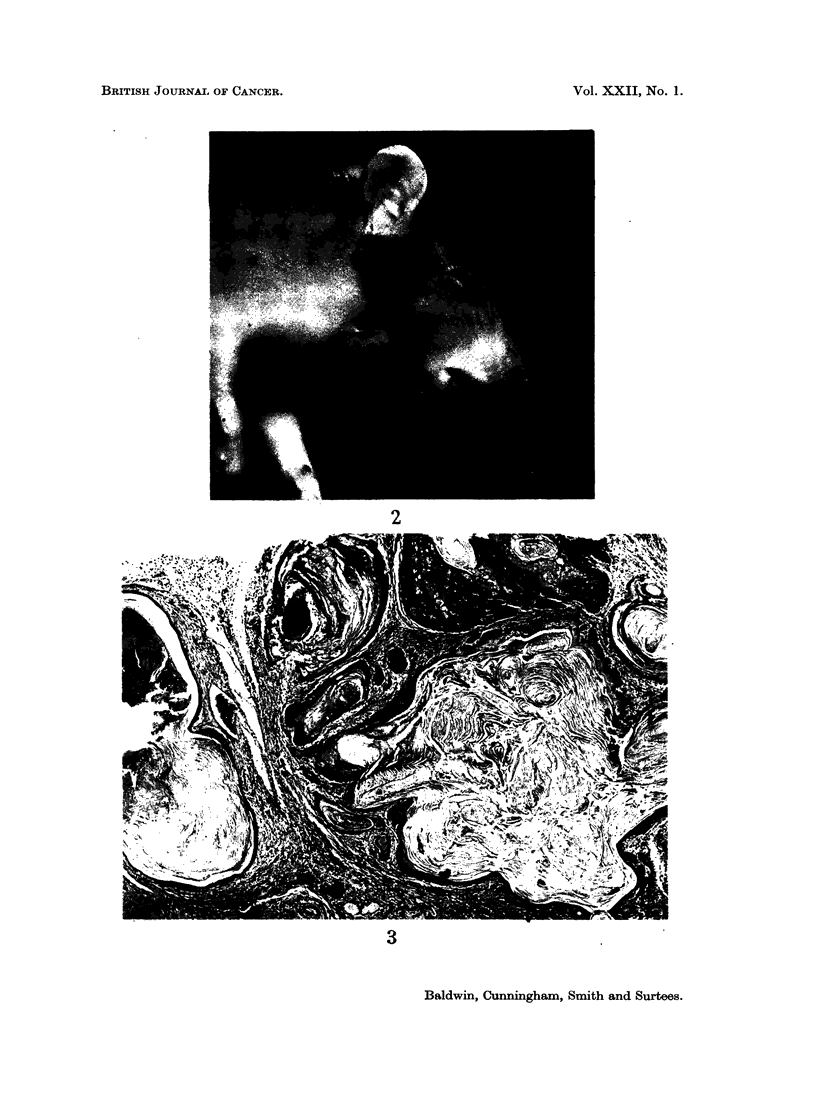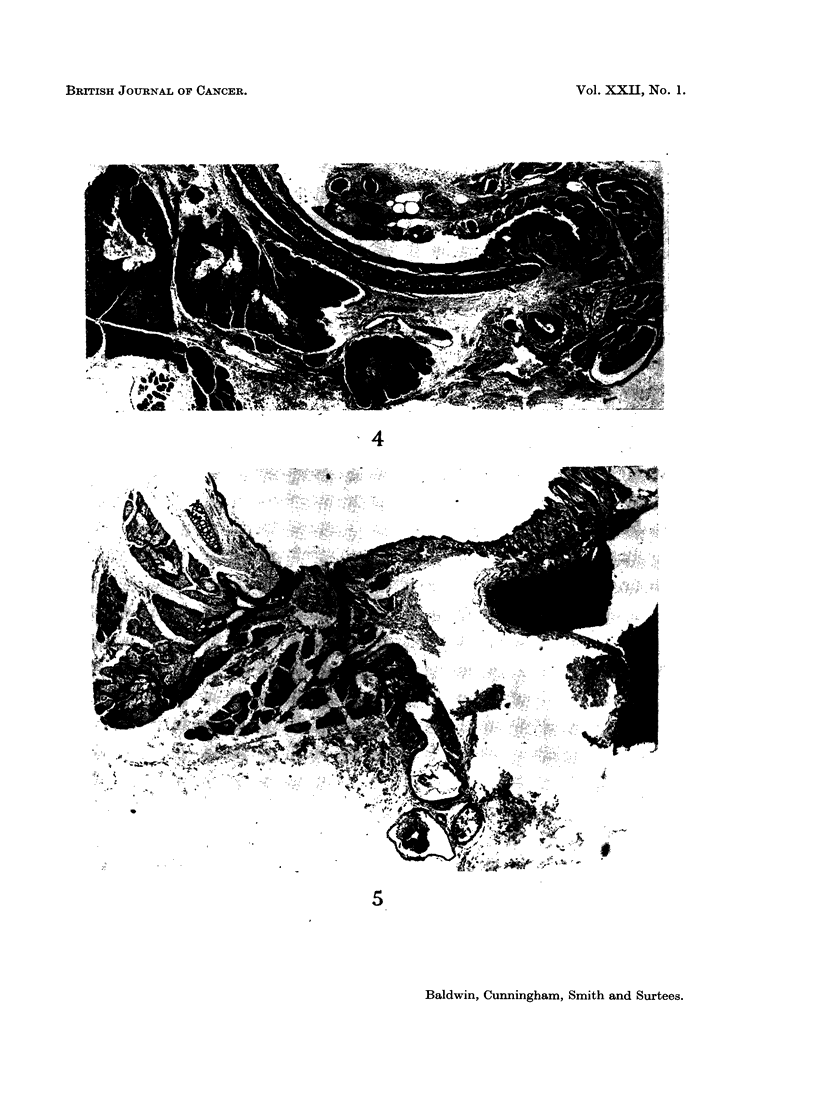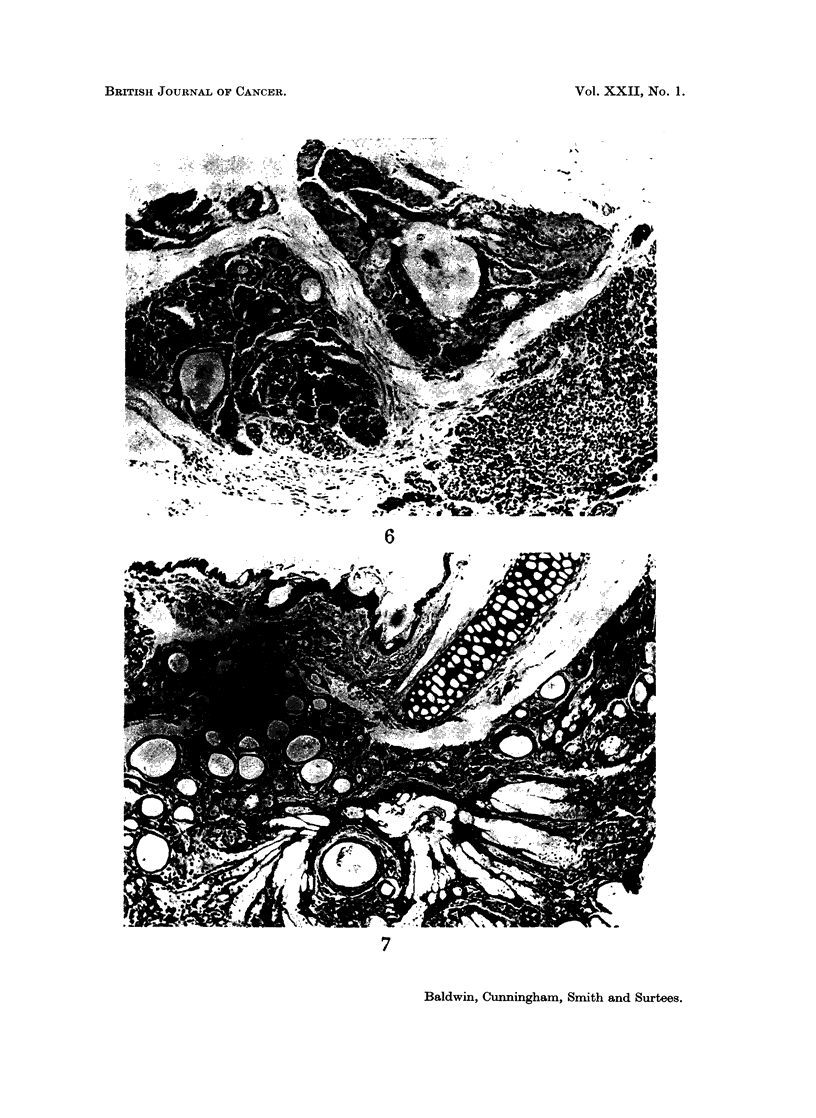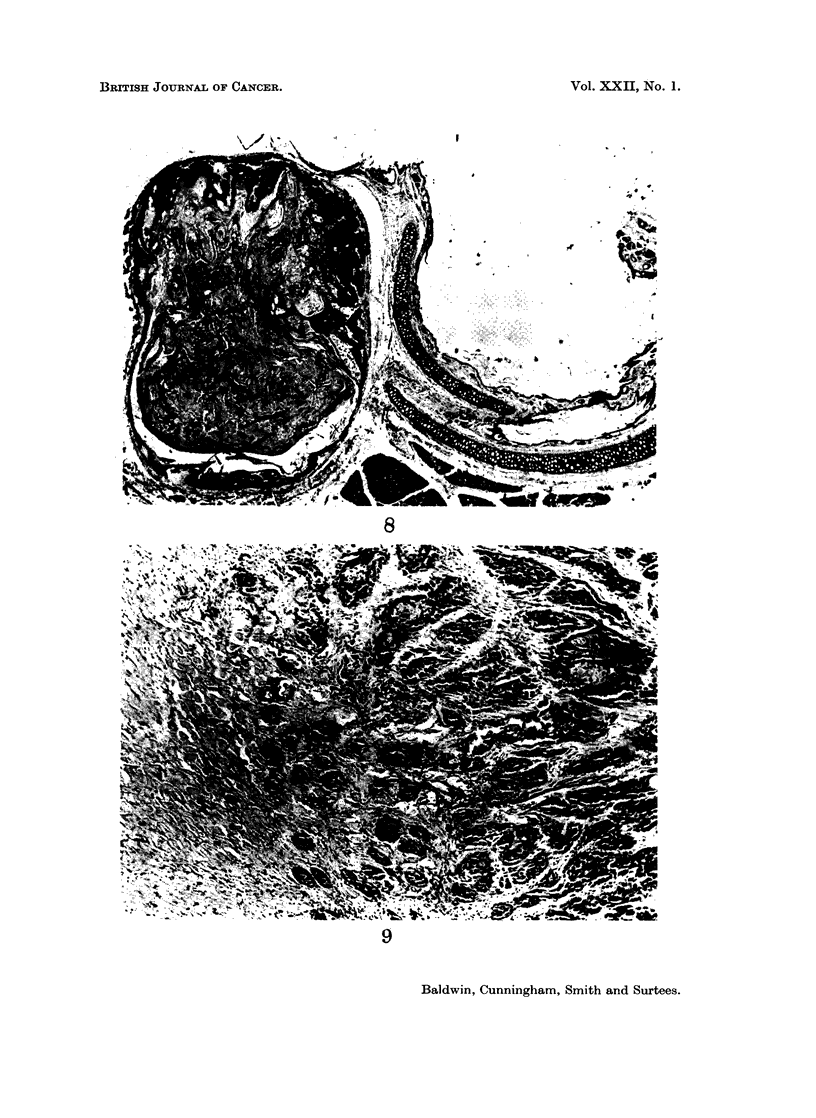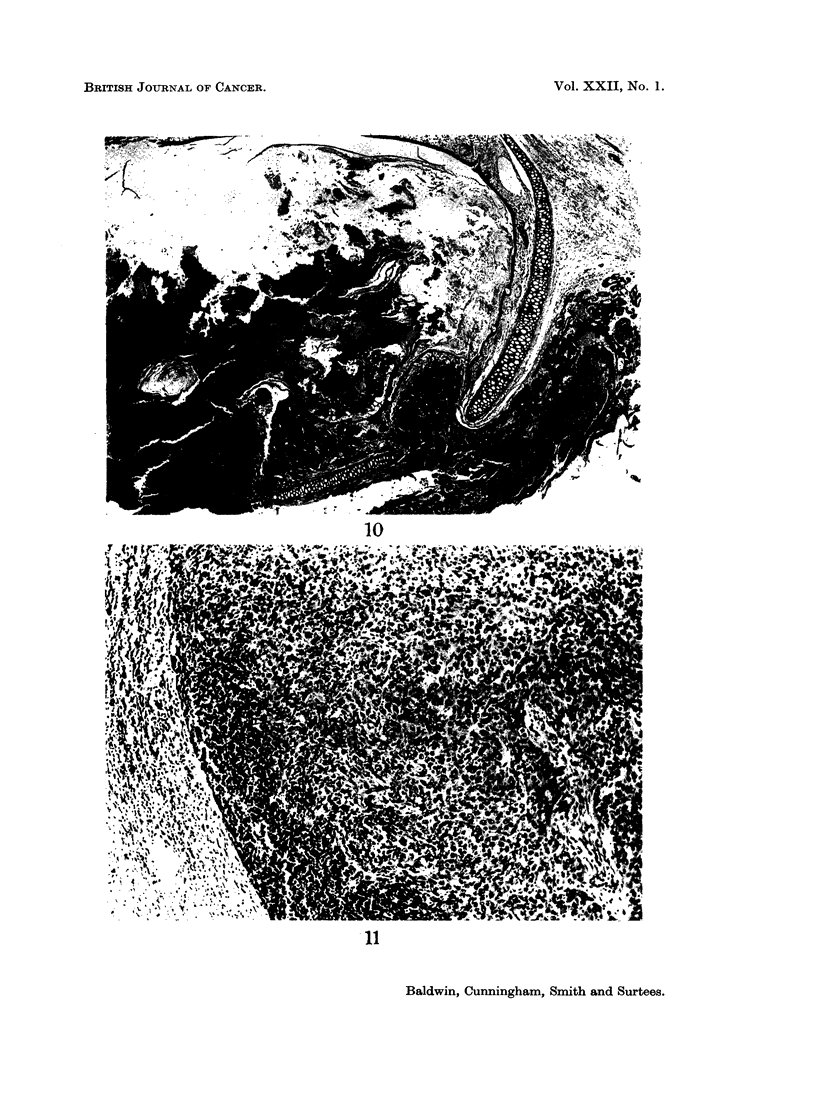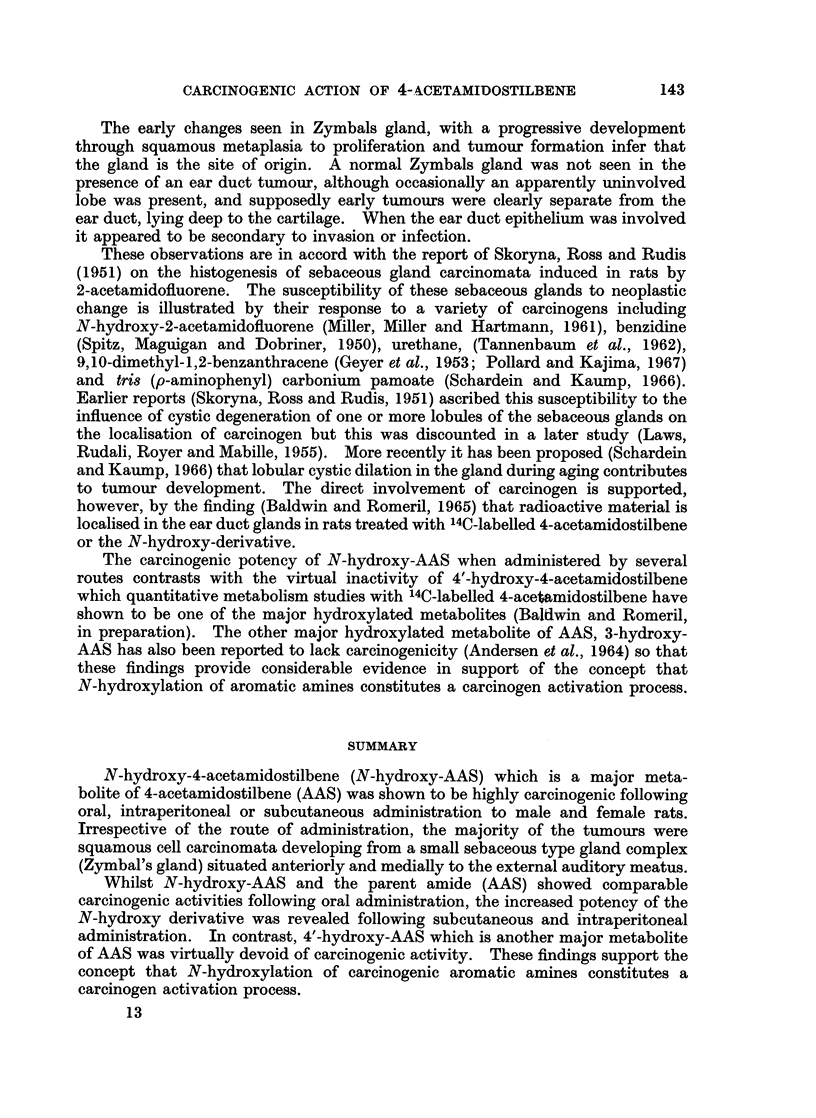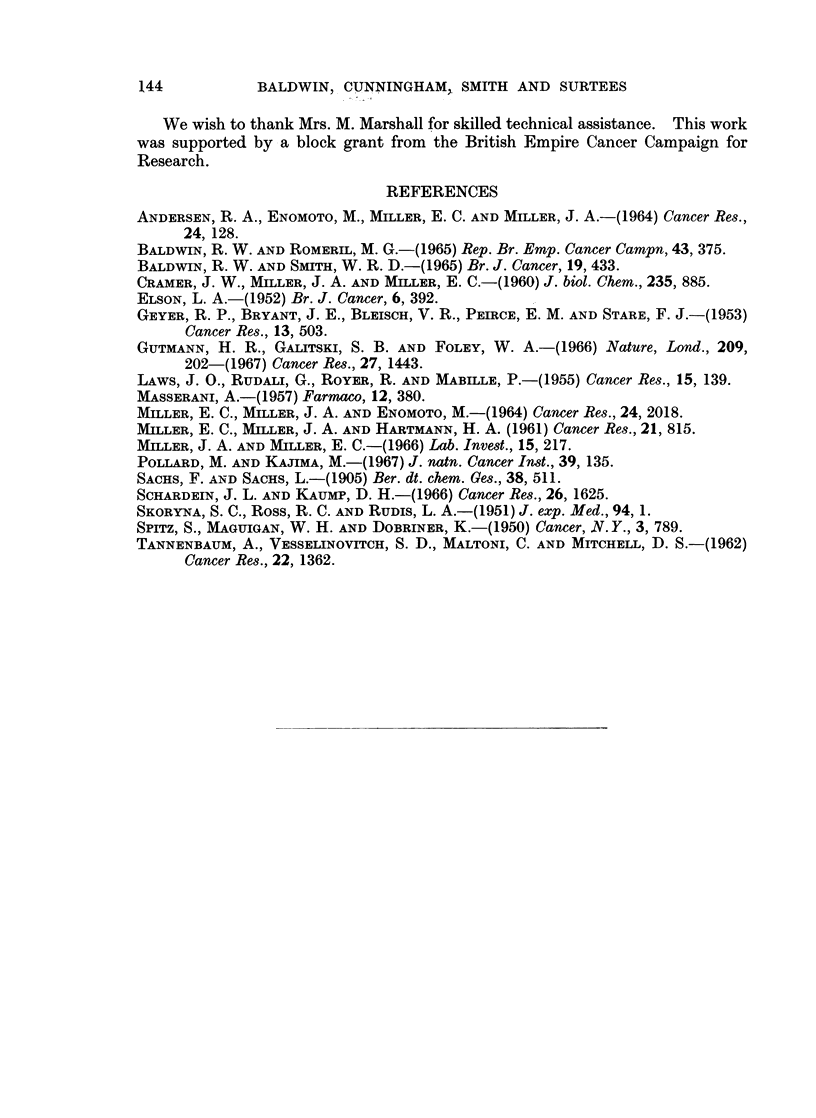# Carcinogenic action of 4-acetamidostilbene and the N-hydroxy derivative in the rat.

**DOI:** 10.1038/bjc.1968.19

**Published:** 1968-03

**Authors:** R. W. Baldwin, G. J. Cunningham, W. R. Smith, S. J. Surtees

## Abstract

**Images:**


### CARCINOGENIC ACTION OF 4-ACETAMIDOSTILBENE AND THE *N*-HYDROXY DERIVATIVE IN THE RAT

R. W. BALDWIN, G. J. CUNNINGHAM, W. R. D. SMITH AND S. J. SURTEES

*From the British Empire Cancer Campaign Research Laboratory, University of Nottingham, and the Department of Pathology, Royal College of Surgeons of England, London*

Received for publication October 27, 1967

*N*-HYDROXYLATED metabolites of several carcinogenic aromatic amines and amides have now been demonstrated and in many cases, the *N*-hydroxy compounds have been shown to have enhanced carcinogenic potency (Miller and Miller, 1966). Thus *N*-hydroxy-2-acetamidofluorene produced neoplasms in the guinea-pig, a species which is normally refractory to the carcinogenic action of 2-acetamidofluorene (Miller, Miller and Enomoto, 1964). Similarly, *N*-hydroxy-2-benzamidofluorene is a potent carcinogen in the rat whereas the parent amide, 2-benzamidofluorene is virtually inactive (Gutmann, Galitski and Foley, 1966, 1967).

Studies on the metabolism of 4-acetamidostilbene in the rat have established that an *N*-hydroxy metabolite is formed, the other hydroxylated metabolites identified being 4′-hydroxy- and 3-hydroxy-4-acetamidostilbene (Andersen *et al.*, 1964; Baldwin and Smith, 1965; Baldwin and Romeril, in preparation). In the present studies, the carcinogenic action of *N*-hydroxy-4-acetamidostilbene in the rat has been examined following administration by several routes and its activities have been compared with those of 4-acetamidostilbene and the 4′-hydroxy derivative.

### EXPERIMENTAL

### *Compounds*

Only the *trans* isomers of 4-aminostilbene and its derivatives were examined. These compounds were prepared by the following methods:

4-*Acetamidostilbene* (AAS) by reduction of 4-nitrostilbene with hydrazine and Raney Ni and acetylation of the resulting amine m.p. 234° C.

4-*Dimethylaminostilbene* (DAS) by the action of benzyl magnesium chloride on *p*-dimethylaminobenzaldehyde (Sachs and Sachs, 1905) m.p. 148° C.

4′-*Hydroxy*-4-acetamidostilbene (4′-hydroxy-AAS) by condensation of *p*-acetamidophenylacetic acid and *p*-hydroxy-benzaldehyde (Masserani, 1957) m.p. 238° C.

*N*-*Hydroxy*-4-*acetamidostilbene* (*N*-hydroxy-AAS) by the partial reduction of 4-nitrostilbene with aluminium amalgam, and acetylation (Baldwin and Smith, 1965) m.p. 200° C.

### *Animals*

Male and female rats of a Wistar strain were used for all tests. These were obtained either from the inbred colony maintained in the department or from commercial breeders.

*Oral administration*

4-Acetamidostilbene and derivatives were incorporated into a semi-synthetic 5 per cent protein diet (Elson, 1952), at a level of 40 mg./kg. and these diets were administered so that the average daily dose of compound was 0·5 mg./rat. All rats were provided with water *ad libitum.* Groups of 16 male rats, initially 190–220 g. in weight, received these diets for 224 days (total dose 112 mg./rat). Female rats, initially 170–180 g. in weight, were treated for 150 days (total dose 75 mg./rat). After carcinogen treatment, rats were maintained on the low protein diet until tests were terminated.

*Subcutaneous injection*

Female rats, initially 150–200 g. in weight, received either 3 or 5 subcutaneous injections at fortnightly intervals of the compounds dissolved in propylene glycol (total doses 37·5 and 62·5 mg./kg. body weight). Control rats were treated with propylene glycol.

*Intraperitoneal administration to weanling rats*

In tests with weanling female rats, initially 6 weeks of age (body weight 50–70 g.), suspensions of the compounds, prepared by homogenisation in balanced salt solution containing gum acacia (1·75 per cent w/v), were injected intraperitoneally thrice weekly. Material was prepared freshly for each treatment and all compounds were injected in a standard volume of 0·4 ml./100 g. body weight.

### RESULTS

*Oral administration*

There was a pronounced loss of body weight in female rats during the first 4 weeks of feeding *N*-hydroxy-4-acetamidostilbene in the low protein diet (daily intake approximately 0·5 mg./rat) and after temporary recoveries, further weight losses were observed (Fig. 1) so that carcinogen administration was stopped after 22 weeks (total dose 75 mg./rat). At this time, 12 of the initial 16 rats were surviving, but a further 6 died before the first tumour was observed (30 weeks). In animals examined early, liver damage was a constant feature. The first evidence of damage was a patchy fatty change mainly in the centrilobular region, then areas of congestion and polymorph infiltration appeared with haemorrhages in the parenchyma which went on to necrosis, usually well delineated, and eventual cyst formation. The cellular infiltrate was usually in the periportal areas but the cystic and haemorrhagic foci appeared irregular in distribution. The cysts often contained weakly positive PAS staining material. Adenomatous hyperplasia was sometimes seen in association with the inflammatory response but no liver tumours were seen and the end picture was of a congested liver showing some areas of fatty change and large cystic spaces lined with simple flat epithelium.

Of the 6 female rats surviving when tumours were first observed (30 weeks), 5 developed squamous carcinomata associated with the external auditory meatus and 1 also had an intestinal adenocarcinoma (Table I). This latter tumour consisted of a mass of irregular acini lined by hyperchromatic columnar cells and containing mucus and polymorphs. It appeared to be a typical large bowel carcinoma.

Male rats tolerated oral administration of *N*-hydroxy-AAS much better than females and there was no loss of body weight during the 32 weeks of treatment (Fig. 1). Tumours were first detected in male rats after 33 weeks and at this time, there were 13 survivors. When the test was terminated after 50 weeks, 12 rats (95 per cent) had developed squamous cell carcinomata in the external auditory meatus. Bilateral ear duct carcinomata were present in 5 rats and 1 also had a fibrosarcoma associated with the gastro-intestinal tract (Table I).

Fig. 1.—Changes in body weight following oral administration to rats of 4-acetamidostilbene (AAS, ○——○) and the *N*-hydroxy derivative (*N*-hydroxy-AAS, □——□) in a low protein diet.

4-Acetamidostilbene was well tolerated following oral administration (Fig. 1) but for comparison with *N*-hydroxy-AAS treated rats, the compound was fed for 22 weeks (total dose 75 mg./rat) and 32 weeks (total dose 112 mg./rat) in female and male rats respectively. Ear duct carcinomata were first detected in these rats between 24 and 26 weeks after the start of treatment at which time all 16 male rats and 13 female rats were surviving. At the completion of the test (48 weeks) ear duct carcinomata had been detected in 11 female and 14 male rats respectively (Table I). Bilateral ear duct carcinomata were detected in 2 of these rats whilst 1 rat with an ear duct carcinoma at one side also had a spindle cell sarcoma in the other ear duct region.

4-Dimethylaminostilbene was also well tolerated when fed in a low protein diet so that the daily intake was approximately 0·5 mg./rat, and the compound

TABLE I.—*Carcinogenic Action of 4-Aminostilbene Derivatives in Rats Following Oral Administration*

| Compound | Initial number of rats | Duration of feeding (days) | Average total dose (mg.) | First tumour observed (weeks) | Number of survivors | Duration of test weeks | Tumour Incidence: Number of rats with tumours | Tumour Incidence: Percentage | Tumours observed* |
|---|---|---|---|---|---|---|---|---|---|
| 4-Dimethylaminostilbene | 16F | 224 | 112 | 28 | 11 | 60 | 6 | 55 | Ear duct carcinoma (6) |
| | 16M | 224 | 112 | 28 | 16 | | 4 | 25 | Ear duct carcinoma (5) |
| 4-Acetamidostilbene | 16F | 150 | 75 | 26 | 13 | 48 | 11 | 85 | Ear duct carcinoma (12); Spindle cell sarcoma (1) |
| | 16M | 224 | 112 | 24 | 16 | | 14 | 88 | Ear duct carcinoma (15); Ear duct papilloma (1) |
| *N*-Hydroxy-4-acetamidostilbene | 16F | 150 | 75 | 30 | 6 | 50 | 5 | 83 | Ear duct carcinoma (5); Intestinal adenocarcinoma (1) |
| | 16M | 224 | 112 | 33 | 13 | | 12 | 95 | Ear duct carcinoma (17); Intestinal fibrosarcoma (1) |
| 4′-Hydroxy-4-acetamidostilbene | 16F | 250 | 125 | 61 | 14 | 74 | 4 | 28 | Lower jaw squamous papilloma (2); Uterine adenocarcinoma (1); Adrenal adenocarcinoma (1) |
| | 16M | 250 | 125 | — | — | | 0 | 0 | |
| None | 20M | | — | — | — | 50 | 0 | 0 | — |

* Figures in parentheses indicate the number of tumours.

was fed to both male and female rats for 32 weeks (total dose 112 mg./rat). Ear duct carcinomata were first detected in these rats after 28 weeks treatment at which time all 16 males and 11 females were surviving. The incidence of ear duct carcinomata was much lower in these rats compared to those obtained with 4-acetamidostilbene or the *N*-hydroxy compound (Table I). Furthermore, females appeared to be more susceptible than males, final tumour incidence in males and females being 25 and 55 per cent respectively.

4′-Hydroxy-4-acetamidostilbene (4′-hydroxy-AAS) which was detected as a major metabolite of AAS and *N*-hydroxy-AAS (Baldwin and Smith, 1965) was almost devoid of carcinogenic activity (Table I). Hence, 29 out of the original 32 rats were surviving without any sign of tumour at 50 weeks when tests with the other compounds were completed. No ear duct tumours were observed in any rats treated with this compound, the only tumours eventually observed being 2 benign papillomata on the lower jaw, a uterine adenocarcinoma and poorly differentiated adenocarcinoma probably originating in the adrenals.

*Subcutaneous injection*

*N*-Hydroxy-AAS produced a marked chronic inflammatory response following three subcutaneous injections (1·25 mg./100 g. body weight) at 2-weekly intervals and 9 of 24 rats were killed within 10 weeks of treatment with chronic abscesses at the injection site. The usual picture was of necrosis and haemorrhage with attempts at the formation of granulation tissue. Individual animals showed simple pyogenic abscesses, others large necrotic areas with more chronic inflammatory changes. In some animals the reparative process was the main feature with a very cellular reaction containing many mitotic figures and bizarre-looking cells. Usually the area was well circumscribed and represented an obvious granulomatous response but in a few animals it was not possible to be certain whether the picture represented extreme hyperplasia or the development of malignancy.

Tumours were first detected 40 weeks after the initial injection and at this time there were 10 survivors. Eight of these rats had developed tumours when the test was terminated after 60 weeks (Table II). Ear duct carcinomata were observed in 6 rats whilst 1 had a squamous cell carcinoma at the injection site and another a mammary adenocarcinoma.

In a second experiment where *N*-hydroxy-AAS was injected 5 times at weekly intervals (total dose, 6·25 mg./100 g. body weight), rats survived much better so that 15 were alive when the first tumour was observed (32 weeks). Six of these rats developed squamous carcinomata associated with the external auditory meatus whilst 1 had a spindle cell sarcoma at the injection site (Table II).

4-Acetamidostilbene was much less toxic than the *N*-hydroxy derivative and all the rats survived 3 subcutaneous injections at 2-weekly intervals (total dose, 3·75 mg./100 g. body weight). At this dose level, only 1 rat developed a tumour, a spindle cell sarcoma at the injection site, during the period of the test (55 weeks).

In the second test when AAS was injected 5 times at weekly intervals (total dose 6·25 mg./100 g. body weight) tumours were observed in only 2 rats (13 per cent). Both these tumours were mammary adenocarcinomata and no ear duct tumours were observed. No tumours were observed in rats treated with propylene glycol which was used as the vehicle for the aminostilbene compounds (Table II).

TABLE II.—*Carcinogenic Action of 4-Aminostilbene Derivatives in Female Rats Following Subcutaneous Injection*

| Compound | Initial number of rats | Dose/ Injection/ 100 g. body wt (mg.) | Number of injections | Total dose mg./100 g. body wt | First tumour observed (weeks) | Number of survivors | Total tumour incidence: Number of rats with tumours | Total tumour incidence: Percentage | Tumour types* |
|---|---|---|---|---|---|---|---|---|---|
| 4-Acetamidostilbene | 20 | 1·25 | 3 | 3·75 | 38 | 17 | 1 | 6 | Spindle cells sarcoma (1) |
| *N*-Hydroxy-4-acetamidostilbene | 24 | 1·25 | 3 | 3·75 | 40 | 10 | 8 | 80 | Ear duct carcinoma (6)<br>Local squamous cell carcinoma (1)<br>Mammary adenocarcinoma (1) |
| 4-Acetamidostilbene | 16 | 1·25 | 5 | 6·25 | 27 | 16 | 2 | 13 | Mammary adenocarcinoma (2) |
| *N*-Hydroxy-4-acetamidostilbene | 16 | 1·25 | 5 | 6·25 | 32 | 15 | 7 | 47 | Ear duct carcinoma (6)<br>Local spindle cell sarcoma (1)<br>Ovarian sarcoma (1) |
| Propylene glycol | 20 | — | 3 | — | — | 20 | 0 | 0 | — |

* Figures in parentheses indicate the number of tumours.

*Intraperitoneal administration to weanling female rats*

*N*-Hydroxy-AAS proved to be highly toxic following repeated intraperitoneal injection into weanling female rats (total dose, 11·2 mg./100 g. body weight) and whilst rats survived the 4-week period of treatment, all but 2 died during the following 12 weeks (Table III, Group II). Almost every animal showed severe intra-abdominal effects. These varied from a few scattered adhesions to areas of necrosis and abscess formation affecting the whole peritoneal cavity and viscera. Sometimes pleural adhesions were also seen. The capsule of the spleen was often thickened, perisplenitis was frequently present with macrophage and small round cell infiltration and occasionally abscesses were seen. Similar changes were found in the liver and the intestines were often stuck down and attached to the anterior abdominal wall. In some animals, chronic inflammation with granulation tissue was present and in the course of the healing process, calcification and lymphoid hyperplasia was seen occasionally.

A similar toxic effect was obtained when the compound was administered at a lower dose level (total dose 6·0 mg./100 g. body weight) and again only 4 out of 16 rats were surviving when tumours were first observed (59 weeks). Of these survivors, 3 developed squamous carcinomata associated with the external auditory meatus but there were no other tumours. In a further experiment where *N*-hydroxy-AAS was administered at a still lower dose (total dose 3·0 mg./100 g. body weight) all but 2 rats were surviving when the first tumour was observed (45 weeks). Ear duct carcinomata were observed in 8 of these rats and another had a papillary adenocarcinoma invading the diaphragm and mesenteric fat giving a total tumour incidence of 64 per cent (Table III).

In contrast, rats tolerated 4-acetamidostilbene administered over 8 injections (total dose 10·0 mg./100 g. body weight) and 13 rats were surviving when the first tumour was detected after 46 weeks. Ear duct carcinomata were observed in 4 of these rats, 1 also having a mammary adenocarcinoma. In addition 2 rats had adenocarcinomata of the large bowel giving a total tumour incidence of 46 per cent (Table III). In a second test where AAS was administered thrice weekly at a dosage of 0·7 mg./100 g. body weight (total dose 11·2 mg./100 g.) all but 1 rat survived until tumours were first observed (36 weeks). Ear duct carcinomata developed in 8 of these rats (tumour incidence 53 per cent) but no other tumours were observed (Table III).

In contrast to the previous report of Andersen *et al.*, (1964) only one of the weanling rats injected intraperitoneally with 4-acetamidostilbene developed a mammary adenocarcinoma. In some cases, changes were seen mainly of a benign nature with varying degrees of ductal hyperplasia and cystic dilatation. The duct usually contained stagnating secretion and retention cysts with some surrounding fibrosis were present in some animals.

*Pathogenesis of ear duct tumour formation*

The early macroscopic sign of abnormality was the appearance of a fixed swelling, often lobulated, below and anterior to the ear duct and pinna (Fig. 2). The mass grew rapidly with distention and eventually ulceration of the skin. No growth was seen to issue from the ear duct itself. A few tumours when fully developed extended almost from the jaw to the axilla but usually the animal was killed or ulceration and infection had supervened before this stage was reached.

TABLE III.—*Carcinogenic Action of 4-Aminostilbene Derivatives Following Intraperitoneal Administration to Weanling Female Rats*

| Compound | Initial number of rats | Dose/ injection/ 100 g. body wt (mg.) | Number of injections | Total dose mg./100 g. body wt | First tumour observed (weeks) | Number of survivors | Tumour incidence: Number of rats with tumours | Tumour incidence: Percentage | Tumour types* |
|---|---|---|---|---|---|---|---|---|---|
| Group I | | | | | | | | | |
| 4-Acetamidostilbene | 16 | 1·25 | 8 | 10·0 | 46 | 13 | 6 | 46 | Ear duct carcinoma (4)<br>Mammary adenocarcinoma (1)<br>Large bowel adenocarcinoma (2) |
| *N*-Hydroxy-4-acetamidostilbene | 16 | 1·25 | 8 | 10·0 | — | 0 | — | — | |
| Gum acacia-saline (controls) | 16 | — | 8 | — | — | 16 | 0 | — | |
| Group II | | | | | | | | | |
| 4-Acetamidostilbene | 16 | 0·7 | 16 | 11·2 | 36 | 15 | 8 | 53 | Ear duct carcinoma (8) |
| *N*-Hydroxy-4-acetamidostilbene | 16 | 0·7 | 16 | 11·2 | — | 0 | — | — | |
| | 16 | 0·5 | 12 | 6·0 | 59 | 4 | 3 | 75 | Ear duct carcinoma (3) |
| | 16 | 0·25 | 12 | 3·0 | 45 | 14 | 9 | 64 | Ear duct carcinoma (8)<br>Peritoneal adenocarcinoma (1) |
| Gum acacia-saline (controls) | 16 | — | 16 | — | — | 16 | 0 | — | |

* Figures in parentheses indicate the number of tumours.

Histologically the tumours showed the typical characteristics of squamous carcinoma the better differentiated showing keratin cysts (Fig. 3). In ear ducts examined before obvious tumours were present it appeared that changes were already present in Zymbals gland. This is a small, sebaceous type gland complex situated anteriorly and medially to the ear duct, deep to the cartilaginous rings almost at the junction of the osseous and cartilaginous portions of the ear duct (Fig. 4). It consists of 3 main lobules and drains into the ear duct through a gap in the cartilage via a duct (Fig. 5), which may be duplicated, lined by flattened or cuboidal epithelium. The ear duct epithelium itself contains simple sebaceous glands.

The first change noticed was dilatation of the lobules with formation of small cysts (Fig. 6). This occurred in an irregular manner, often commencing in only 1 section of the gland but soon involving the ducts throughout their length. Sometimes the lobular dilatation spread to involve all the gland (Fig. 7) but in general 1 or 2 lobules only were affected. The process proceeded to gross dilatation of the duct and lobules, the lumen of the space being filled with the debris of necrotic cells and the epithelial lining appeared to undergo squamous metaplasia. Eventually the secretory epithelium was replaced by stratified squamous cells with considerable keratin formation which was desquamated into the lumen.

The mass of squamous epithelium sometimes developed papillary formations into the centre of the tumour and by further growth formed a circumscribed keratin cyst with a pseudocapsule of compressed surrounding tissue. At this stage it could be clearly seen that the ear duct was not involved and that the tumour was deep to the cartilage and a short distance away from the ear duct (Fig. 8). Other tumours were less well demarcated with the cystic areas and tumour cells extending into the neighbouring tissue. The cartilaginous rings were often found surrounded by tumour and appeared necrotic but there was no direct invasion of the cartilage or bone. Occasionally, the appearances resembled actual invasion of the ear duct through gaps in the cartilage as shown in Fig. 10. Some tumours were of a particularly invasive nature and showed extensive infiltration of muscle (Fig. 9). Secondary deposits in the lymph nodes of the neck were found in a few animals (Fig. 11).

Zymbals gland lies in close association with the salivary glands and part of the lacrimal gland. No tumour of these glands was found but, changes were seen in association with neighbouring tumour masses; round cell infiltration and haemorrhages were not uncommon and sometimes the glands appeared disorganised with some of the nuclei enlarged and of bizarre shape.

### DISCUSSION

Following the discovery by Cramer, Miller and Miller (1960) of *N*-hydroxy-2-acetamidofluorene as a urinary metabolite of 2-acetamidofluorene in the rat, it has been established in several studies that *N*-hydroxy derivatives of carcinogenic aromatic amines are also carcinogenic often with increased activity or a reactivity to a wider range of tissues (see review by Miller and Miller, 1966). The present studies demonstrating the high carcinogenicity of *N*-hydroxy-4-acetamidostilbene add to the body of evidence indicating that *N*-hydroxylation is a critical metabolic transformation.

Both $N$-hydroxy-AAS and the parent amide produced a high incidence of ear duct carcinomata following oral administration so that it was not possible under these conditions to assess the relative potencies of the 2 compounds. However, 4′-hydroxy-4-acetamidostilbene which constitutes another major metabolite of both $N$-hydroxy-AAS and AAS (Baldwin and Smith, 1965; Baldwin and Romeril, in preparation) was virtually devoid of activity when administered orally and none of the rats treated with this compound developed ear duct carcinomata (Table I). In comparable studies, Andersen *et al.*, (1964) observed a high incidence of ear duct carcinomata in male rats orally treated with $N$-hydroxy-AAS or the parent amide. $N$-Hydroxy-AAS but not AAS also induced tumours of the gastro-intestinal tract. These latter findings differ from those obtained in the present study where only 2 rats treated with $N$-hydroxy-AAS developed intestinal tumours, and possibly reflect variations in the tissue susceptibilities of the rat strains used in the two studies.

The enhanced carcinogenicity of $N$-hydroxy-AAS compared with that of the parent amide was revealed more convincingly following subcutaneous injection of the compounds (Table II). Thus tumour incidences of 47 per cent and 80 per cent were obtained in 2 tests with $N$-hydroxy-AAS, the majority of the tumours being ear duct carcinomata. Comparable tests with AAS produced few tumours (tumour incidences 6 per cent and 13 per cent) and none were ear duct carcinomata. The greater activity of $N$-hydroxy-AAS compared with AAS following subcutaneous injection of the compounds into rats was also reported by Andersen *et al.*, (1964) but in these studies the tumours induced by $N$-hydroxy-AAS were predominantly mammary carcinomata and no ear duct tumours were detected. The low susceptibility of mammary tissue in the Wistar rat strain used in the present studies was further demonstrated by the almost complete lack of response at these sites to $N$-hydroxy-AAS or AAS when administered intraperitoneally to weanling female rats (Table III). The majority of tumours induced by this treatment were ear duct carcinomata and again the greater activity of the $N$-hydroxy-AAS was evident. These results contrast with those of Andersen *et al.*, (1964) who observed a 43 per cent incidence of mammary tumours in immature female rats treated in a similar manner with $N$-hydroxy-AAS.

The greatly increased incidence of tumours in treated animals compared with controls leaves little doubt that the aminostilbene compounds were the carcinogenic agents in the production of these tumours.

---

### EXPLANATION OF PLATES

FIG. 2.—Gross appearance of established ear duct tumour.

FIG. 3.—Well differentiated tumour of low malignancy, showing many keratin cysts. H. and E. ×37.

FIG. 4.—Zymbals gland. The ear duct epithelium, with simple sebaceous glands, is also shown separated from the gland by cartilage. H. and E. ×15.

FIG. 5.—Zymbals gland, with slight dilatation of one of the lobes, to show the opening into the ear duct at the junction of the osseous and cartilaginous portions. H. and E. ×15.

FIG. 6.—Part of Zymbals gland showing the sebaceous nature of the cells and early cyst formation. H. and E. ×90.

FIG. 7.—Cyst formation and dilatation affecting most of the gland. H. and E. ×90.

FIG. 8.—A circumscribed tumour lying some distance from the ear duct, which appears normal. H. and E. ×15.

FIG. 9.—Showing infiltration of muscle. H. and E. ×90.

FIG. 10.—Apparent invasion of the ear duct through gaps in the cartilage. H. and E. ×15.

FIG. 11.—Secondary deposits in a lymph node. H. and E. × 90.

2

3

Baldwin, Cunningham, Smith and Surtees.

4

5

6

7

Baldwin, Cunningham, Smith and Surtees.

8

9

Baldwin, Cunningham, Smith and Surtees.

10

11

Baldwin, Cunningham, Smith and Surtees.

The early changes seen in Zymbals gland, with a progressive development through squamous metaplasia to proliferation and tumour formation infer that the gland is the site of origin. A normal Zymbals gland was not seen in the presence of an ear duct tumour, although occasionally an apparently uninvolved lobe was present, and supposedly early tumours were clearly separate from the ear duct, lying deep to the cartilage. When the ear duct epithelium was involved it appeared to be secondary to invasion or infection.

These observations are in accord with the report of Skoryna, Ross and Rudis (1951) on the histogenesis of sebaceous gland carcinomata induced in rats by 2-acetamidofluorene. The susceptibility of these sebaceous glands to neoplastic change is illustrated by their response to a variety of carcinogens including *N*-hydroxy-2-acetamidofluorene (Miller, Miller and Hartmann, 1961), benzidine (Spitz, Maguigan and Dobriner, 1950), urethane, (Tannenbaum *et al.*, 1962), 9,10-dimethyl-1,2-benzanthracene (Geyer *et al.*, 1953; Pollard and Kajima, 1967) and *tris* (*ρ*-aminophenyl) carbonium pamoate (Schardein and Kaump, 1966). Earlier reports (Skoryna, Ross and Rudis, 1951) ascribed this susceptibility to the influence of cystic degeneration of one or more lobules of the sebaceous glands on the localisation of carcinogen but this was discounted in a later study (Laws, Rudali, Royer and Mabille, 1955). More recently it has been proposed (Schardein and Kaump, 1966) that lobular cystic dilation in the gland during aging contributes to tumour development. The direct involvement of carcinogen is supported, however, by the finding (Baldwin and Romeril, 1965) that radioactive material is localised in the ear duct glands in rats treated with $^{14}$C-labelled 4-acetamidostilbene or the *N*-hydroxy-derivative.

The carcinogenic potency of *N*-hydroxy-AAS when administered by several routes contrasts with the virtual inactivity of 4′-hydroxy-4-acetamidostilbene which quantitative metabolism studies with $^{14}$C-labelled 4-acetamidostilbene have shown to be one of the major hydroxylated metabolites (Baldwin and Romeril, in preparation). The other major hydroxylated metabolite of AAS, 3-hydroxy-AAS has also been reported to lack carcinogenicity (Andersen *et al.*, 1964) so that these findings provide considerable evidence in support of the concept that *N*-hydroxylation of aromatic amines constitutes a carcinogen activation process.

### SUMMARY

*N*-hydroxy-4-acetamidostilbene (*N*-hydroxy-AAS) which is a major metabolite of 4-acetamidostilbene (AAS) was shown to be highly carcinogenic following oral, intraperitoneal or subcutaneous administration to male and female rats. Irrespective of the route of administration, the majority of the tumours were squamous cell carcinomata developing from a small sebaceous type gland complex (Zymbal's gland) situated anteriorly and medially to the external auditory meatus.

Whilst *N*-hydroxy-AAS and the parent amide (AAS) showed comparable carcinogenic activities following oral administration, the increased potency of the *N*-hydroxy derivative was revealed following subcutaneous and intraperitoneal administration. In contrast, 4′-hydroxy-AAS which is another major metabolite of AAS was virtually devoid of carcinogenic activity. These findings support the concept that *N*-hydroxylation of carcinogenic aromatic amines constitutes a carcinogen activation process.

We wish to thank Mrs. M. Marshall for skilled technical assistance. This work was supported by a block grant from the British Empire Cancer Campaign for Research.

### REFERENCES

ANDERSEN, R. A., ENOMOTO, M., MILLER, E. C. AND MILLER, J. A.—(1964) *Cancer Res.*, **24**, 128.
BALDWIN, R. W. AND ROMERIL, M. G.—(1965) *Rep. Br. Emp. Cancer Campn*, **43**, 375.
BALDWIN, R. W. AND SMITH, W. R. D.—(1965) *Br. J. Cancer*, **19**, 433.
CRAMER, J. W., MILLER, J. A. AND MILLER, E. C.—(1960) *J. biol. Chem.*, **235**, 885.
ELSON, L. A.—(1952) *Br. J. Cancer*, **6**, 392.
GEYER, R. P., BRYANT, J. E., BLEISCH, V. R., PEIRCE, E. M. AND STARE, F. J.—(1953) *Cancer Res.*, **13**, 503.
GUTMANN, H. R., GALITSKI, S. B. AND FOLEY, W. A.—(1966) *Nature, Lond.*, **209**, 202—(1967) *Cancer Res.*, **27**, 1443.
LAWS, J. O., RUDALI, G., ROYER, R. AND MABILLE, P.—(1955) *Cancer Res.*, **15**, 139.
MASSERANI, A.—(1957) *Farmaco*, **12**, 380.
MILLER, E. C., MILLER, J. A. AND ENOMOTO, M.—(1964) *Cancer Res.*, **24**, 2018.
MILLER, E. C., MILLER, J. A. AND HARTMANN, H. A. (1961) *Cancer Res.*, **21**, 815.
MILLER, J. A. AND MILLER, E. C.—(1966) *Lab. Invest.*, **15**, 217.
POLLARD, M. AND KAJIMA, M.—(1967) *J. natn. Cancer Inst.*, **39**, 135.
SACHS, F. AND SACHS, L.—(1905) *Ber. dt. chem. Ges.*, **38**, 511.
SCHARDEIN, J. L. AND KAUMP, D. H.—(1966) *Cancer Res.*, **26**, 1625.
SKORYNA, S. C., ROSS, R. C. AND RUDIS, L. A.—(1951) *J. exp. Med.*, **94**, 1.
SPITZ, S., MAGUIGAN, W. H. AND DOBRINER, K.—(1950) *Cancer, N.Y.*, **3**, 789.
TANNENBAUM, A., VESSELINOVITCH, S. D., MALTONI, C. AND MITCHELL, D. S.—(1962) *Cancer Res.*, **22**, 1362.